# Keeping Dr. Charles Richard Drew’s legacy alive

**DOI:** 10.5195/jmla.2019.726

**Published:** 2019-07-01

**Authors:** Darlene Parker-Kelly, Charles P. Hobbs

**Affiliations:** Director of Library/Learning Resource Center, Health Sciences Library, Charles R. Drew University, Los Angeles, CA, darleneparkerkelly@cdrewu.edu; Librarian, Health Sciences Library, Charles R. Drew University, Los Angeles, CA, charleshobbs@cdrewu.edu

## Abstract

The Charles R. Drew University of Medicine and Science (CDU) recently celebrated its fiftieth anniversary. The university was established to honor Dr. Charles Richard Drew, a pioneer in blood banking. As a tribute to the legacy of CDU and Dr. Drew, the CDU Health Sciences Library examined how CDU is keeping Dr. Drew’s legacy alive.

In 2016, the Charles R. Drew University of Medicine and Science (CDU) celebrated its fiftieth anniversary. This truly was a time of celebration of its existence for over a half of a century and its two minority designations: the only historically black graduate institution west of the Mississippi River and a charter member of the Hispanic-Serving Health Professions Schools. Hence, the CDU Health Sciences Library reflected on the university’s history, looking for an opportunity of discourse by telling a story from our perspective.

Compared with the other three historically black medical schools (Howard University, Meharry College, and Morehouse School of Medicine), CDU is a mere youngster. But to those who have ever attended CDU, worked at CDU, or been a staff member or patient at the King Drew Medical Center, these five decades bring to mind quite a story. This is a story of a medical school and a health care facility born of the civil unrest in the Watts neighborhood of South Central Los Angeles in 1965. To fully understand the historical significance of the university, we must examine Dr. Charles R. Drew, his legacy, and the university.

## WHO WAS DR. CHARLES R. DREW?

Charles R. Drew was born on June 3, 1904, in Washington, DC. He attended Dunbar High School and Amherst College, after which he went to medical school at McGill University in Canada. After completing his internship and residency at Canadian hospitals, he returned to Washington, DC, to work at Howard University and Columbia University on fellowships. He began his research in storing human blood for transfusions and completed his dissertation, *Banked Blood*, in 1940. During World War II, he developed processes for storing blood and moving it to where it was needed: the battlefield.

After his work on blood banking during the war and up until his untimely death on April 1, 1950, Dr. Drew concentrated on teaching the next generation of African-American physicians as a distinguished professor of surgery at Howard University [1]. According to a plaque in the CDU Cobb Building rotunda, “Dr. Drew’s students loved him, therefore a ‘Drew Lecture’ was one not to be missed. When word got out that Dr. Charles Drew was planning to make his rounds to discuss his cases, every intern and resident would be present if at all possible.”

A quick review of an online “white pages” website shows over fifty elementary schools, high schools, health clinics, and other institutions, including our very own CDU, named after the pioneering African-American physician who developed the modern blood banking techniques that are still in use today. How many people sign up with the Red Cross and donate blood, or perhaps are in the unfortunate position to need a blood donation, think about Dr. Drew? A few might know about his struggle with the Red Cross to desegregate the blood supply. Others might know the story of his death and the inaccurate story surrounding it: after his auto accident, he was not refused access to a hospital [2].

The National Library of Medicine (NLM) honors Dr. Drew (one of the few African Americans so honored) on its “Profiles in Science” web page [3]. This site contains links to books, articles, and other materials by and about Dr. Drew. There is even a link to his high school yearbook entry, in which he expressed a desire to be an electrical engineer. Fortunately for the human race, destiny had other plans. In addition, NLM honors Dr. Drew through its traveling exhibit, “Opening Doors: Contemporary African American Academic Surgeons.” Dr. Drew is featured in the pioneers’ section of the exhibit [4]. Interestingly, there are several physicians in this exhibit who were taught by Dr. Drew, and so the legacy continues.

## THE CHARLES R. DREW UNIVERSITY (CDU)

The Charles R. Drew Medical Society (CRDMS) was founded in 1948 as a Los Angeles–based affiliate of the National Medical Society [5]. Although the CRDMS and other community leaders in South Central Los Angeles had advocated for a local hospital since the early 1950s, nothing was done until after the 1965 Watts Revolt. The McCone Commission, which was formed by California State Governor Pat Brown in response to the civil unrest, cited lack of access to health care as one of the underlying causes. Following the commission’s recommendation, a new hospital was planned, built, and opened in 1972 as the Martin Luther King Hospital [6].

Before the hospital opened, the CRDMS advocated for and founded a medical school, to be known as the Charles R. Drew Postgraduate Medical School, in 1966 [7]. The hospital and medical school became known, jointly, as the “King Drew Medical Center” in 1982 [8].

## THE CDU HEALTH SCIENCES LIBRARY AND DR. DREW’S LEGACY

Beyond naming a building or institution after him, how do we keep Dr. Drew’s legacy alive? While we cannot speak for the other institutions named after him, we can discuss how the CDU and its Health Sciences Library continue his legacy.

At CDU, when one walks into the Cobb building (containing the College of Medicine, the Health Sciences Library, and university administration), one sees photographs, newspaper articles, samples of work, and other memorabilia related to Dr. Drew. With some imagination and the efforts of the Health Sciences Library, there is a combined vision to continue Dr. Drew’s legacy.

The founding library director was M. Moss Humphrey, who established a library that serves our students, faculty, staff, and persons in the community [9]. Ms. Humphrey established the CDU Archives, which preserves historical papers, photos, and other memorabilia about CDU and Dr. Drew. The library has begun digitizing these historical documents. In addition, the library has a collection of books about Dr. Drew, including a copy of his dissertation *Banked Blood*, and makes every effort to acquire new titles about him. The library also provides access to several articles by and about Dr. Drew in print, online, or via interlibrary loan. These articles can be accessed by visiting the CDU Dr. Drew LibGuide [10].

**Figure 1:**
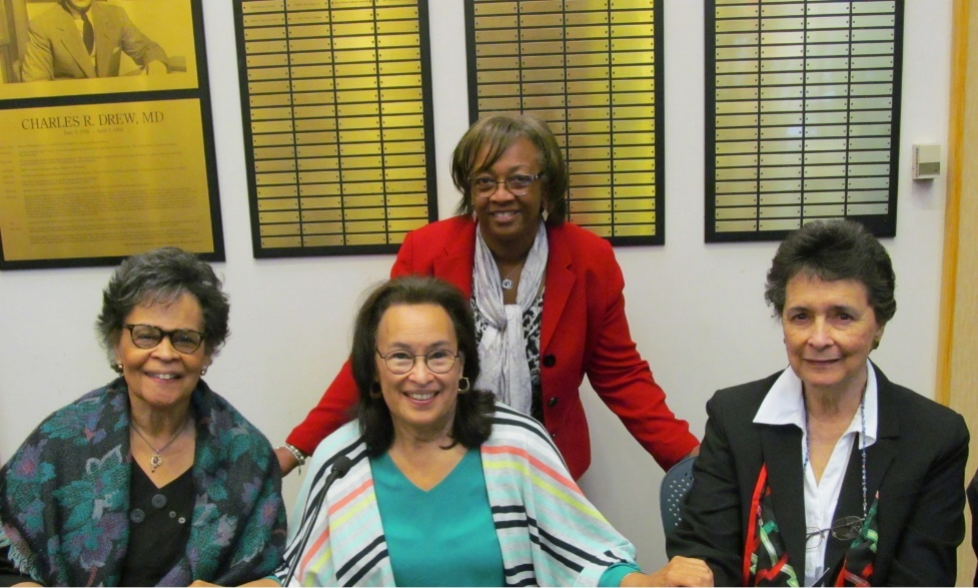
The Drew siblings: Charlene Drew Jarvis, Sylvia Drew Ivie, and Bebe Drew Price (sitting); Darlene Parker-Kelly, director, Health Sciences Library, Charles R. Drew University of Medicine and Science (CDU) (standing)

In May 2017, the current library director collaborated with Dr. Drew’s youngest daughter, Sylvia Drew Ivie, to have all three of his daughters in a panel discussion about their father (Figure 1). It was during this dialogue that everyone in attendance learned much more about Dr. Drew’s love for mankind and that, even though his children were young, they understood that he held great responsibility. During this panel discussion, Dr. Drew’s family brought him to life, and we believe those in attendance will forever be grateful for obtaining greater insight into Dr. Drew’s life and legacy.

One of the outstanding processes that have taken place at CDU is the “President’s Freshman Seminar” course, during which students learn more about Dr. Drew from readings and through conversation with Dr. Drew’s daughter. The library contributes to this course by securing select biographies on Dr. Drew and assisting with copyright.

The authors thought it would be fitting to ask Sylvia Drew Ivie to join us in this History Matters narrative. We asked her several questions, and we provide excerpts from the interview transcripts.

### Can you share with us one of your fondest memories of your father, Dr. Charles R. Drew?

“My fondest memories of him are planting flowers. We had a rental house on the campus of Howard University, and there was a backyard, where he had planted flowers. He liked to come home from work and go immediately into the backyard and tend to the flowers.

“He allowed me to help him plant a little red plant called statice when I was very little, and he was very methodical about it. He would dig the hole, and he would put the plant in it, and it was my job to pull the dirt up around it and to pat it tight; we couldn’t go on to the second flower until I had done a good job on the first one, and so we would stay with the first one until it was to his satisfaction.

**Figure 2:**
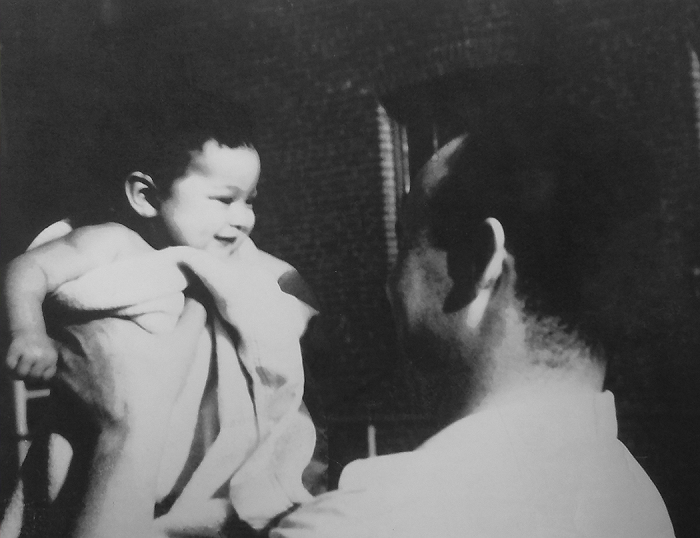
Sylvia Drew Ivie and Dr. Charles R. Drew in front of their apartment on Sherman Avenue, Washington DC, 1944

“That was really who my father was; he wanted every effort to be done at a level of excellence, and he was patient but insistent that it had to be done in that way. I didn’t feel that he was angry with me because I had to be instructed, but it was clear that we were not going to be moving on until each one was done the proper way” (Figure 2).

### How do you think we can preserve Dr. Drew’s legacy?

“Well, first of all, I think it’s a very, very great honor that there is a school in his name because we do not have a way of remembering so many of the great African-American contributors to this country and to our people. If you memorialize such individuals in a living institution, then the person has a chance of not being forgotten.”

### How well does the CDU preserve the legacy of Dr. Drew? Is there anything it can do differently to improve this effort?

“I think the very existence of this university in his name keeps the memory of him alive with just the bricks, the mortar, and the example of his life keeps his memory in front of people for generations today and generations to come. The university being named in my dad’s honor is a terrific gift to everyone who knew him, and also for our esteemed students, faculty, staff, and visitors. I think the university does a wonderful job of preserving his legacy through pictures and various educational programming opportunities. For example, in the spring of 2017, the University honored our family at the University’s Spring Gala, and our library sponsored ‘Conversations with Dr. Charles R. Drew’s Heirs,’ which included a dialogue with my sisters, Bebe Drew Price, Charlene Drew Jarvis, PhD, and me as the facilitator. This event allowed us to share memories of our father and to bring him to life for many of the attendees” (Figure 1).

“There were many instances where my family collaborated with CDU to preserve the legacy of my father, Dr. Drew. I recall very earlier on when my mother came out to cut the ribbon on the first mobile trailer that the University owned west of the school. At that time there was only the trailer which housed the University and we continue to preserve those early documents through the University Archives. Interestingly, one [of] my father’s student from Howard University Dr. Mitchell Spellman became the first dean of CDU and knew him intimately, admired him, and shared stories about his life. There were several affiliations between the faculty here at CDU who were first at Howard University where my father taught. I believe it is a continuation process and we encourage our students to learn about the wonderful contributions that my father made. Also, we want our students to realize their abilities and their potential for even greater contributions to the field [of] biomedical sciences. The aspect that I think might be done in furtherance of his legacy is to try to distill his mind a little more, try to distill his thinking a little more, and toward that end, and when I speak to medical students, I remind them of [the] importance of understanding the whole person.”

### What do historically black colleges and universities (HBCUs) offer to students and faculty that makes them different?

“When I reflect on the importance of Historically Black College and Universities (HBCUs), although we’re separated by a whole country, we are connected to one another. We have one dream of educating and advancing the under-resourced through education.

“Well, it’s a terrible thing not to be seen, it’s a terrible thing to be ignored, it’s a terrible thing to be given a message that you are other and not important and none of those things happen at HBCUs.

“You feel in this environment that whoever you are and whatever you can be is wanted and is believed in. The presumption is that you have talent, the presumption is that you’re capable of excellence. That presumption has not historically existed in other schools.”

### How do HBCUs vary amongst themselves?

“Well, I think we [CDU] don’t have the depth of history that Howard University has. We haven’t been around 150 years and I think that hurts us in terms of alumni and foundation support, also in terms of finances because Howard gets federal dollars that we don’t get, but I think the fact that we’re newer gives us an advantage because the values that we live by are more inclusive of other under-resourced communities and their leaders.

“We have values that are shown in our development of a community faculty, for example, are shown in our requirement of knowing something about international health, shown in our values of being multi-ethnic, happily multi-ethnic, in our composition. I think we have taken the best cultural elements of the original HBCUs and just added another layer to the cake. We are progressive politically because we’re younger and it’s perhaps a generational difference.”

### Will HBCUs be needed in the future? What might their role be?

“Well, who could have anticipated Donald Trump, who could have anticipated that we would be at this juncture in our life as a nation after all of the struggle that we’ve been through to make a place of respect for ourselves and here someone who in two years’ time has made it perfectly alright to disrespect, disavow, take away from everything that we’ve worked to establish. There’s no telling how far back this is going to take us, there’s no telling.

“This University is an infrastructure holding of all that we believe, all that we’ve strived to establish. This is the place where our humanity and our brilliance and our excellence will continue to be demonstrated, proved, and utilized in the training of excellent providers and breakthrough research, and so we will be needed indefinitely.”

## CONCLUSION

Upon further reflection, we indeed have a story to tell, and it is “history matters.” Our histories are more important than ever as we prepare for the next decade. We seek to encourage others to see the connections, the legacies, and the possibilities. We examined our university and discussed select means that we employ to keep Dr. Drew’s legacy alive. Additionally, the library has identified a valuable niche that we can continue to explore all the more.

## 

**Darlene Parker-Kelly**, darleneparkerkelly@cdrewu.edu, Director of Library/Learning Resource Center, Health Sciences Library, Charles R. Drew University, Los Angeles, CA

**Charles P. Hobbs**, charleshobbs@cdrewu.edu, Librarian, Health Sciences Library, Charles R. Drew University, Los Angeles, CA
